# DNA Methylation Profiling of Breast Cancer Cell Lines along the Epithelial Mesenchymal Spectrum—Implications for the Choice of Circulating Tumour DNA Methylation Markers

**DOI:** 10.3390/ijms19092553

**Published:** 2018-08-28

**Authors:** Anh Viet-Phuong Le, Marcin Szaumkessel, Tuan Zea Tan, Jean-Paul Thiery, Erik W. Thompson, Alexander Dobrovic

**Affiliations:** 1 Olivia Newton John Cancer Research Institute, Heidelberg, VIC 3084, Australia; anh.vple@gmail.com (A.V.-P.L.); Marcin.Szaumkessel@onjcri.org.au (M.S.); 2 Department of Surgery, St. Vincent’s Hospital, University of Melbourne, Melbourne, VIC 3065, Australia; e2.thompson@qut.edu.au; 3 Cancer Science Institute of Singapore, 14 Medical Drive, National University of Singapore, Singapore 117599, Singapore; csittz@nus.edu.sg (T.Z.T.); bchtjp@nus.edu.sg (J.-P.T.); 4 INSERM UMR 1186, Integrative Tumor Immunology and Genetic Oncology, Gustave Roussy, Université Paris-Sud, 94805 Villejuif, France; 5 Institute of Health and Biomedical Innovation and School of Biomedical Sciences, Queensland University of Technology, Kelvin Grove, QLD 4059, Australia; 6 Translational Research Institute, Woolloongabba, QLD 4102, Australia; 7 Department of Clinical Pathology, University of Melbourne, Parkville, VIC 3010, Australia; 8 School of Cancer Medicine, La Trobe University Bundoora, Bundoora, VIC 3086, Australia

**Keywords:** DNA methylation, epithelial–mesenchymal plasticity, breast cancer, methylation-sensitive high-resolution melting (MS-HRM), pyrosequencing, biomarkers, minimal residual disease, circulating tumour DNA

## Abstract

(1) Background: Epithelial–mesenchymal plasticity (EMP) is a dynamic process whereby epithelial carcinoma cells reversibly acquire morphological and invasive characteristics typical of mesenchymal cells. Identifying the methylation differences between epithelial and mesenchymal states may assist in the identification of optimal DNA methylation biomarkers for the blood-based monitoring of cancer. (2) Methods: Methylation-sensitive high-resolution melting (MS-HRM) was used to examine the promoter methylation status of a panel of established and novel markers in a range of breast cancer cell lines spanning the epithelial–mesenchymal spectrum. Pyrosequencing was used to validate the MS-HRM results. (3) Results: *VIM*, *DKK3*, and *CRABP1* were methylated in the majority of epithelial breast cancer cell lines, while methylation of *GRHL2*, *MIR200C*, and *CDH1* was restricted to mesenchymal cell lines. Some markers that have been used to assess minimal residual disease such as *AKR1B1* and *APC* methylation proved to be specific for epithelial breast cell lines. However, *RASSF1A*, *RARβ*, *TWIST1*, and *SFRP2* methylation was seen in both epithelial and mesenchymal cell lines, supporting their suitability for a multimarker panel. (4) Conclusions: Profiling DNA methylation shows a distinction between epithelial and mesenchymal phenotypes. Understanding how DNA methylation varies between epithelial and mesenchymal phenotypes may lead to more rational selection of methylation-based biomarkers for circulating tumour DNA analysis.

## 1. Introduction

Analysis of circulating tumour DNA (ctDNA) is increasingly being used for the monitoring of minimal residual disease (MRD) during cancer treatment. Detection of ctDNA is dependent on the use of cancer-specific markers [1]. As breast cancer has few recurrent single site mutations outside of those in the *PIK3CA* gene [2], attention is being shifted to DNA methylation, in which a relatively small panel of markers might be applicable to the majority of tumours.

As DNA methylation markers have been reported to differ across the epithelial–mesenchymal spectrum, the identification of a panel of DNA methylation markers that take epithelial–mesenchymal plasticity (EMP) into account would be desirable. EMP refers to the dynamic transition across the epithelial–mesenchymal axis. This is a crucial event during normal development, and one of the developmental mechanisms that cancer cells take advantage of in order to disseminate [3]. Epithelial carcinoma cells can undergo epigenetic alterations, changing their morphology and behaviour to become more migratory and invasive [4,5,6,7]. Through epithelial–mesenchymal transition (EMT), these cells can escape from the tumour into the tissues and circulation and travel to secondary sites, where they lead to the formation of metastases that appear to have undergone mesenchymal–epithelial transition [8]. Tumours and their metastases have been shown to consist of heterogeneous mixtures of epithelial and mesenchymal cells reflecting epithelial–mesenchymal plasticity [9,10].

We sought to examine how the methylation of both established and novel markers varied across the epithelial and mesenchymal states in a panel of breast cancer cell lines that encompass the epithelial–mesenchymal spectrum. Our hypothesis was that relating the locus-specific methylation status to epithelial and mesenchymal states would enable clarity in the choice of DNA methylation markers for monitoring MRD using ctDNA. To our knowledge, this is the first study that characterizes the DNA methylation status of a panel of breast cancer cell lines spanning the epithelial–mesenchymal spectrum. Examining DNA methylation across the epithelial–mesenchymal spectrum is a novel approach to identify optimal DNA methylation markers for ctDNA. 

## 2. Results

### 2.1. Ranking of Breast Cancer Cell Lines across the Epithelial–Mesenchymal Spectrum

**Figure 1:**
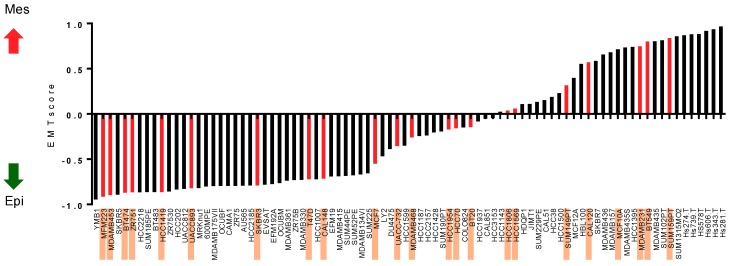
The alignment of breast cancer cell lines across the epithelial–mesenchymal spectrum. Breast cancer cell lines are placed from the most epithelial (lowest EMT score; *y*-axis) to the most mesenchymal (highest EMT score) in the epithelial–mesenchymal spectrum. Breast cancer cell lines included for DNA methylation analysis in this study are highlighted and correspond to the red bars on the graph. The red and black arrows indicate increasing mesenchymal scores and increasing epithelial scores respectively.

In order to relate the DNA methylation status to epithelial and/or mesenchymal phenotypes, we first ranked the twenty-six breast cancer cell lines on the epithelial–mesenchymal spectrum using a previously published quantitative scoring system [11]. Epithelial cell lines have negative scores on this scale, with MFM-223 being the most epithelial of the cell lines (Figure 1). Positive scores indicate mesenchymal cell lines, with Hs578T being the most mesenchymal of the cell lines (Figure 1). The MDA-MB-468, HCC1954, HCC70, BT-20, HCC1806, HCC1569, SUM-149PT, and CAL-120 cell lines were considered to have intermediate epithelial–mesenchymal phenotypes [11]. 

Data to rank the PMC42 cell line (PMC42-ET) and its more mesenchymal sub-line (PMC42-LA) [12] were not available. PMC42-ET and PMC42-LA were placed within the basal B group based on independent clustering of gene expression data (Blick, Tomaskovic-Crook, Neve, Thompson; unpublished data).

Table 1 summarises the hormonal receptor status, vimentin status, molecular subgroup, and epithelial and mesenchymal status of these cell lines based on publicly available datasets and publications. The data for the expression of the estrogen receptor (ER), progesterone receptor (PR), and human epidermal growth factor receptor 2 (HER2) of selected cell lines were found in the American Type Culture Collection (ATCC) website and references therein [13]. For the cell lines whose hormone receptor status could not be found in the ATCC, this information was extracted from Kao et al. [14] and Lehmann et al. [15], as indicated in Table 1. Vimentin expression was previously determined by us [16,17]. Breast cancer cell line intrinsic subgroups, including luminal, basal A, and basal B, were according to the Neve et al. [18], Hoeflich et al. [19], and Heiser et al. [20] gene expression studies. HER2-amplification status in MDA-MB-453, BT-474, HCC1419, UACC-893, HCC1954, HCC1569, and SK-BR-3 cell lines, reported in the GSE12790 data by Hoeflich et al. [19], was confirmed by Kalous et al., using fluorescence in situ hybridization (FISH) [21]. We observed that the majority of luminal and HER2-amplified cell lines are epithelial, whereas basal B and triple negative (ER−/PR−/HER2−) cell lines are mesenchymal. Basal A cell lines usually have an intermediate phenotype, regardless of HER2 status.

### 2.2. The DNA Methylation Status of the Breast Cancer Cell Lines

**Figure 2:**
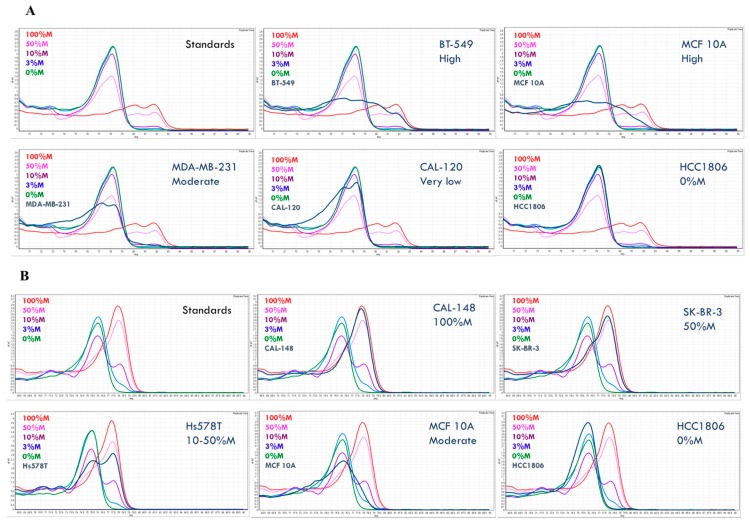
Methylation-sensitive high-resolution melting (MS-HRM) results of representative samples for (**A**) *GRHL2* and (**B**) *RASSF1A*. Both homogeneous methylation and heterogeneous methylation can be seen. Methylation level was numerically scored for samples with homogeneous methylation, whereas it was called ‘high’, ‘moderate’, and ‘very low’ for a high level, moderate level, and very low level of heterogeneous methylation, respectively.

To determine the methylation status for the sixteen genes, we employed methylation-sensitive high-resolution melting (MS-HRM), which amplifies regions of interest regardless of methylation status and enables the differentiation of methylated, unmethylated, and partially methylated templates after the polymerase chain reaction (PCR) by high-resolution melting (HRM) analysis [22,23]. A series of methylation standards was included in each MS-HRM run to estimate the methylation level in the samples. By comparing the melting profiles of the amplified products from the MS-HRM assays to the 0% and 100% controls, it can be determined if methylation is homogeneous or heterogeneous (Figure 2A,B).

The melting profiles of the methylation standard series exemplify homogeneous methylation, in which CpG sites within the same amplicons are collectively methylated or unmethylated. The PCR products from the methylated templates with higher melting temperature have melt peaks to the right, while the PCR products from the unmethylated templates melt earlier, and thus have melt peaks to the left. A sample with a melting profile resembling that of the methylation standards is homogeneously methylated. In this case, the methylation level can be semi-quantified using the methylation standard series. For example, HCC1806 is fully unmethylated for *GRHL2* (Figure 2A), CAL-148 is fully methylated, and SK-BR-3 is homogeneously methylated at 50% for *RASSF1A* (Figure 2B).

When the methylation profile extends across either or both sides of the fully unmethylated control peak, the sample is heterogeneously methylated. Heterogeneous methylation patterns result as a consequence of heteroduplex formation from multiple different partially methylated templates within the same sample [24]. Figure 2A gives examples of heterogeneous *GRHL2* methylation in the BT-549, MCF-10A, MDA-MB-231, and CAL-120 cell lines. Figure 2B shows an example of heterogeneous *RASSF1A* methylation in the MCF 10A cell line.

In contrast to homogeneous methylation, the level of heterogeneous methylation is less readily determined by visual examination due to the complexity of the methylation patterns. We scored the heterogeneous methylation level as very high, high, moderate, low, or very low based on the degree to which the melting curves extend into the fully methylated profiles. The further the melting curves extend under the fully methylated peak, the higher the overall methylation levels (e.g., Figure 2A,B).

The PCR products were further assessed by bisulfite pyrosequencing to determine the average methylation percentage at each CpG site for the selected markers, in order to obtain additional information about methylation and to validate the MS-HRM results [25]. Due to the possible bias introduced by MS-HRM assays at given temperatures, (i.e., overestimation or underestimation of methylation levels), the methylation standards were also pyrosequenced together with the samples. Table 2 shows pyrosequencing data for the methylation standard series and representative samples of each available methylation level calling for *GRHL2* methylation and *RASSF1A* methylation.

The pyrosequencing results for *GRHL2* (Table 2A) and *RASSF1A* (Table 2B) indicate that the estimation of methylation levels by MS-HRM is well matched with the values given by pyrosequencing. The degree of extension into the fully methylated profile is consistent with the levels of methylation overall. Pyrosequencing was also performed for other markers, including *AKR1B1*, *RARβ*, *SFRP2*, *GFRA1*, and *CRABP1* (Figure S1 in Supplementary Materials). The concordance between the methylation level estimation and the pyrosequencing data of the samples relative to those of the methylation standard series supported our estimates of the methylation levels for heterogeneous methylation made directly from the melting curves.

### 2.3. *Heterogeneous Methylation Is Frequent for the Studied Markers*

The methylation results for the studied markers in the breast cancer cell line panel are presented in Table 3. Samples with homogeneous methylation were marked with numerical values for their methylation levels, whereas those with heterogeneous methylation are marked as ‘very high’, ‘high’, ‘moderate’, ‘low’, and ‘very low’ as an estimation of their methylation levels, concordant with pyrosequencing results.

Some methylation markers commonly used for monitoring breast cancer, including *APC*, *RASSF1A*, and *RARβ*, predominantly displayed patterns consistent with homogeneous methylation. Other less commonly used markers for monitoring, such as *AKR1B1* and *SFRP2*, showed both homogeneous and heterogeneous methylation patterns in the cell line samples. In contrast, the majority of the other markers showed heterogeneous methylation patterns. This was most pronounced for the mesenchymally methylated markers: *GRHL2*, *MIR200C*, and *CDH1*. In addition, the majority of the mesenchymal cell lines displayed heterogeneous methylation patterns in most genes compared to the epithelial cell lines.

### 2.4. Methylation Patterns and EMP Status in Breast Cancer Cell Lines

Since methylation at the promoter region generally silences gene expression, methylation of markers that are typically expressed in epithelial cells, i.e., *GRHL2*, *MIR200C*, and *CDH1*, was considered potentially mesenchymal-specific, and methylation of markers that are typically expressed in mesenchymal cells, i.e., *TWIST1*, *DKK3*, *VIM*, *CRABP1*, and *EGFR*, was considered potentially epithelial-specific. In addition, we assessed several DNA methylation markers that have not yet been associated with epithelial or mesenchymal status, but have been proposed for the monitoring of MRD (*AKR1B1*, *APC*, *RASSF1A*, *RARβ*, *SFRP2*, and *GFRA1*).

In Table 3, the genes in this study are grouped into epithelial expression-based (epithelial group), mesenchymal expression-based (mesenchymal group), or those previously used as minimal residual disease markers for which the epithelial or mesenchymal status remains to be determined (EMP-unassigned group). The methylation result of each cell line for each gene marker is colour-coded, with red being 100% homogeneous methylation or very high heterogeneous methylation; orange being 50% homogeneous methylation or high heterogeneous methylation; yellow being 10–50% homogeneous methylation or moderate heterogeneous methylation; light yellow being less than 10% homogeneous methylation or low heterogeneous methylation; and white being less than 3% homogeneous methylation, no methylation, or very low heterogeneous methylation. To compensate for non-specific background, methylation levels less than 3% for homogeneous methylation and the very low heterogeneous methylation group were ignored, although they may still represent true minor cellular subpopulations.

The mesenchymally-expressed markers *DKK3*, *CRABP1*, and *VIM* were methylated at moderate to high levels in the majority of epithelial cell lines. Several epithelial cell lines were fully methylated for these genes; for example, MFM-223 for *CRABP1*, and MFM-223 and MDA-MB-453 for *DKK3.* The methylation of *DKK3* and *CRABP1* was not restricted to epithelial cell lines, but was also seen in some intermediate and mesenchymal cell lines (Table 3)*. DKK3* was methylated in the epithelial cell lines BT-20, MCF-7, T-47D, and ZR-75-1 and in the mesenchymal cell line MDA-MB-231, but not in Hs578T, similar to the findings of Veeck et al. [26]. SK-BR-3 is the only epithelial cell line that was not methylated for *DKK3*.

In contrast, *VIM* was methylated in most epithelial cell lines except for BT-474. While the majority of intermediate and mesenchymal basal cell lines were unmethylated for *VIM*, the BT-20 and MDA-MB-231 cell lines were methylated at 100% and 10%, respectively. Interestingly, the archetypal mesenchymal cell line MDA-MB-231 also displayed moderate to high methylation levels for the mesenchymal markers *DKK3* and *CRABP1*, in contrast to the other mesenchymal cell lines, which may indicate a higher plasticity of this cell line.

*EGFR* was strongly methylated in only a few epithelial cell lines compared to the other mesenchymal markers. Three epithelial cell lines, i.e., MDA-MB-453, T-47D, and UACC-732, showed relatively moderate to high methylation levels, with UACC-732 being methylated at a high level for *EGFR*. The methylation of *EGFR* in MDA-MB-453 is consistent with the study of Montero et al. [27]. All the mesenchymal cell lines had no or very low methylation for this marker, consistent with its expression in mesenchymal cell lines.

*TWIST1* was methylated across the epithelial and mesenchymal spectrum from low to very high levels. The methylation distribution of *TWIST1* was different from other mesenchymally expressed markers, the methylation of which was predominantly seen in epithelial cell lines.

*RARβ* was also methylated in both epithelial and mesenchymal cell lines, with the methylation level being higher in epithelial cell lines, except for HCC1419 and T-47D. Most mesenchymal cell lines were also methylated at moderate to high levels for *RARβ*, with the exceptions of Hs578T, which had no methylation, and MDA-MB-231 and BT-549, which had a very low level of methylation.

Similar to the methylation distribution of *RASSF1A* and *RARβ*, *SFRP2* was methylated across the epithelial and mesenchymal spectrum, except for a few cell lines including HCC70, HCC1806, BT-549, and Hs578T. Its methylation level was moderate to high in the majority of cell lines, in both epithelial and mesenchymal states. Surprisingly, the intermediate cell lines BT-20 and HCC1569 displayed the highest methylation level (100%) for *SFRP2*.

*GFRA1* was methylated in fewer epithelial, intermediate, and mesenchymal cell lines, compared to other genes in the EMP-unassigned group. Interestingly, it was also fully methylated in the intermediate cell lines BT-20 and HCC1569, as seen for *SFRP2* methylation, and the epithelial cell line MFM-223.

The other cancer-associated markers *APC* and *AKR1B1* showed relatively high methylation levels in epithelial cell lines, whilst no methylation was observed in mesenchymal cell lines. Two exceptions for *APC* were the epithelial cell lines SK-BR-3, with low methylation, and HCC1419 and T-47D, with no methylation.

The majority of cell lines did not amplify for *CDKN2A.* Among those, MCF-7, MDA-MB-231, BT-20, and Hs578T were reported to have a homozygous deletion [28]. The T-47D and HCC1954 cell lines were homogeneously methylated at 100%, the MFM-223 cell line was moderately methylated, and the ZR-75-1 and HCC1569 cell lines were methylated at low levels for *CDKN2A.* All the breast cancer cell lines tested were unmethylated for *BRCA1*.

**Figure 3:**
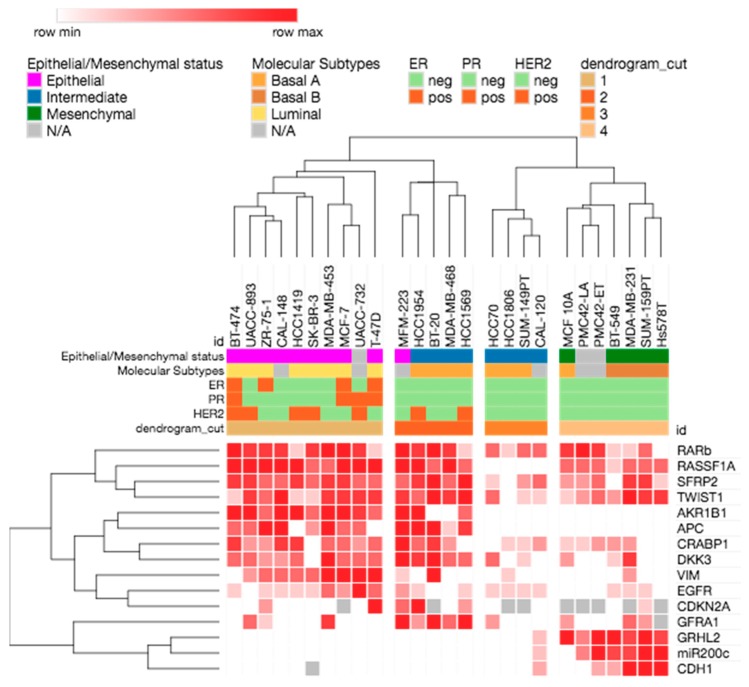
Unsupervised hierarchical clustering of the breast cancer cell lines according to methylation of the selected gene markers.

Unsupervised hierarchical clustering of all the breast cancer cell lines for all the gene markers gave rise to four main clusters (Figure 3). The methylation profiles separated the cell lines according to epithelial and mesenchymal phenotypes, and also the intrinsic subtypes. Cluster 1 contained predominantly epithelial cell lines of luminal subtype. Cluster 2 was comprised of both luminal and basal A cell lines. Cluster 3 had mainly basal A intermediate cell lines, while cluster 4 was comprised of all the mesenchymal basal B and triple-negative breast cancer cell lines. Most ER+, PR+, and HER2+ cell lines fell into clusters 1 and 2.

## 3. Discussion

In this study, we determined the methylation status of 16 gene markers in a panel of 26 breast cancer cell lines spanning the epithelial–mesenchymal spectrum. This is of relevance for selecting an optimal panel of methylation markers to assess MRD in breast cancer, given that ctDNA may derive from tumour cells across the epithelial–mesenchymal spectrum, as well as circulating and disseminated tumour cells, which can also contribute to the ctDNA. Our study also provides some insight on the role of DNA methylation in the molecular stratification of tumours.

A panel of methylation-based markers, the expression of which is associated with mesenchymal or epithelial phenotypes, were selected and grouped into mesenchymally-expressed (i.e., *TWIST1*, *DKK3*, *VIM*, *CRABP1*, and *EGFR*) and epithelially-expressed (i.e., *GRHL2*, *MIR200C*, and *CDH1*) markers, respectively. Methylation of most promoter regions is associated with a lack of gene expression. Thus, when a gene is predominantly expressed in mesenchymal cells, its methylation would be expected to be predominantly epithelial. Our results showed a close-to-expected distribution of the epithelial methylation and mesenchymal methylation markers along the epithelial–mesenchymal spectrum.

The majority of mesenchymal methylation markers were indeed predominantly methylated in epithelial cell lines, while the epithelial methylation markers were methylated in mesenchymal cell lines. All the epithelial and intermediate cell lines were unmethylated for *GHRL2*, *MIR200C*, and *CDH1*, whereas all the mesenchymal cell lines were methylated at moderate to high levels for *GHRL2* and *MIR200C.*

Three mesenchymal cell lines, MDA-MB-231, SUM-159PT, and Hs578T, were methylated at moderate levels for *CDH1*, consistent with the previous report of Lombaerts et al., who also showed partial methylation in mesenchymal breast cancer cell lines [29]. SK-BR-3 did not show amplification in our assay for *CDH1*, consistent with the report of a promoter deletion by Lombaerts et al. [29].

*GRHL2* expression has been shown to be elevated in epithelial luminal cells and downregulated in mesenchymal breast cancer cell lines [30,31,32]. We showed that epithelial breast cancer cells have no *GRHL2* methylation, whereas mesenchymal basal cell lines displayed moderate to high methylation levels for this marker. This is consistent with findings of the differential *GRHL2* methylation between epithelial and mesenchymal cell lines in non small cell lung cancer [33].

In our study, *MIR200C* was methylated at moderate to high level in mesenchymal breast cancer cell lines. Similarly, Davalos et al. showed methylation of the CpG island associated with the MiR-200 cluster including *MIR200C* in mesenchymal MDA-MB-231 cells, whereas this was unmethylated in epithelial MCF-7 cells [34]. Neves et al. demonstrated that the expression of the miR200c/141 cluster was regulated by DNA methylation, and that *MIR200C* promoter hypermethylation was tightly correlated with mesenchymal phenotype and invasive capacity in a panel of eight breast cancer cell lines [35]. In addition, the loss of MiR-200 expression was found in the stem-cell-like fractions from metastatic breast cancer, and miR200c/141 cluster repression by DNA methylation was associated with the transition from a nonstem to stem-like phenotype in human mammary epithelial (HMLE) cells [36].

Another interesting observation from this study was the methylation status of *EGFR*. *EGFR* is often highly expressed in mesenchymal and basal cell lines [2,20], and thus *EGFR* was expected to be heavily methylated in epithelial luminal cell lines. However, unlike other epithelially methylated markers, *EGFR* was only methylated at moderate to high levels in the two HER2-amplified epithelial cell lines, i.e., MDA-MB-453 and UACC-732, and the HER2-normal epithelial cell line T-47D (Table 1). Interestingly, the HER2-amplified cell lines MDA-MB-453 and UACC-732 are resistant to the pan-HER receptor tyrosine kinase inhibitor dacomitinib, which selectively targets EGFR, HER2, and HER4, whereas the majority of HER2-amplified cell lines were sensitive to the drug [21]. The HER2-normal T-47D cell line [19,37] also showed resistance to dacomitinib [21].

DNA methylation markers previously used in monitoring MRD, including the commonly used *RASSF1A*, *APC*, *TWIST1*, and *RARβ*, and the less commonly used *AKR1B1*, *SFRP2*, *GFRA1*, *BRCA1*, and *CDKN2A*, were also assessed, as the knowledge of how their methylation relates to epithelial–mesenchymal status is relevant to their use as MRD markers. From an MRD perspective, a marker that is methylated across the EMP spectrum will be most useful.

*RASSF1A* methylation was distributed widely across the epithelial–mesenchymal spectrum. Promoter methylation of the *RASSF1A* gene is the most common cause of the gene’s inactivation and is associated with tumour invasion and metastasis [38,39]. We also observed that the methylation level was higher in epithelial cell lines. Most epithelial luminal cell lines have complete methylation, consistent with a previous report that *RASSF1A* hypermethylation was significantly more common in ER-positive and HER2-positive tumours [40].

*TWIST1* promoter methylation has been reported to be a useful marker to detect breast cancer [41]. *TWIST1* showed methylation across the epithelial–mesenchymal spectrum. Figure 3 shows that it clusters with *RASSF1A*, *RARβ*, and *SFRP2*. Although *TWIST1* is considered a master regulator of EMT, DNA methylation may not be the main mechanism that regulates *TWIST1* expression in mesenchymal cells. Indeed, it has been shown that *TWIST1* methylation did not correlate with either mRNA or protein expression in tumours [41].

*RARβ* was methylated at some level in every cell line except Hs578T. Although its methylation was very low in a subset of both epithelial and mesenchymal cell lines, it still proved to be one of the most useful markers in terms of coverage across the EMT spectrum.

*SFRP2* also showed a distribution of methylation across the epithelial–mesenchymal spectrum [42]. Its methylation has been detected at high frequency in cancer patients, typically in lung cancer and gastric cancer [43,44,45]. It is a canonical Wnt pathway inhibitor and has been reported to be a promising novel therapeutic marker for several tumor types [42,44,46]. The loss of *SFRP2* expression interferes with the growth of mammary epithelial cells [42]. *SFRP2* was found to promote epithelial cell transformation and to stimulate cell adhesion to the extracellular matrix in breast tumours, which consequently promotes resistance to apoptosis [47].

*APC* and *AKR1B1* are methylated only in the epithelial cell lines and in some intermediate cell lines. *APC* is an inhibitor of the Wnt signaling pathway that acts by inactivating β-catenin [48,49,50]. Wnt signaling has been implicated in EMP [51], consistent with the selective methylation of *APC* in epithelial cell lines. *AKR1B1* has also been shown to be involved in EMT, where its overexpression is associated with the upregulation of EMT markers in lens epithelial cells [52] and the selective absence of *AKR1B1* methylation in mesenchymal cell lines, consistent with the potential role in breast cancer of EMT recently reported in basal breast cancers [53].

Overall, the methylation data confirmed the general distinction between epithelial and mesenchymal cell lines. By looking at the methylation status of the markers in Table 3, it is possible to distinguish between epithelial cell lines and mesenchymal cell lines. Epithelial cell lines are methylated for the majority of previously used cancer markers in MRD and mesenchymal markers, whereas mesenchymal cell lines are methylated for the epithelially expressed markers.

Epithelial cell lines display higher methylation levels than mesenchymal cell lines, and the methylation seen in the mesenchymal cell lines is often heterogeneous. This observation in cell lines reflects the results from recent studies showing that luminal breast tumours have overall higher methylation levels than other subtypes [54,55,56]. The lower methylation levels of these epithelial markers is perhaps explained by the previous observation that basal cell lines seem to repress genes largely by histone H3K27 methylation in preference to DNA methylation [57,58], and that H3K27 methylation is associated with lower level of DNA methylation [59].

Some interesting patterns were seen for the markers that have been commonly used to monitor MRD. Almost all epithelial cell lines are fully methylated for *RASSF1A* at high levels, whilst most mesenchymal cell lines are also methylated, but to a lesser extent. However, cell lines with an intermediate epithelial–mesenchymal phenotype show a more complex methylation status, and the mesenchymal cell line BT-549 and most of the intermediate cell lines, including HCC70, HCC1806, SUM-149PT, and CAL-120, are unmethylated for *RASSF1A.*

Cell lines with intermediate epithelial–mesenchymal phenotypes show a more complex methylation status of the studied genes, which might reflect their plasticity. A recent study by Chung et al. [58] suggested that cells with the intermediate epithelial phenotype have a looser control of gene expression, and as a consequence, more plasticity than the epithelial cells in ovarian cell type.

By taking advantage of MS-HRM to visualize complex methylation patterns based on the melting profiles, our study revealed that only the markers used commonly to monitor MRD (i.e., *APC*, *RASSF1A*, and *RARβ*) displayed homogeneous methylation overall, while the majority of the other markers showed heterogeneous methylation (Table 3). Previous genome-wide methylation studies have shown that the DNA methylation profiles of breast cancer cell lines largely mirror those of primary tumours [60,61]. Based on the methylation in breast cancer cell lines, it can be predicted that tumour samples may also predominantly show heterogeneous methylation for these studied genes.

Genes with homogeneous methylation are more easily detected with current methodologies compared to those with heterogeneous methylation, especially in the MRD context, due to the simultaneous methylation at each CpG [62]. Our observation in terms of homogeneous and heterogeneous methylation profiles might help to explain why the methylation of *APC*, *RASSF1A*, and *RARβ* genes with a homogeneous pattern have been detected at high frequency and are used successfully in detecting various tumour types as well as MRD. Therefore, we consider that homogeneously methylated markers are more useful for the detection and monitoring of MRD. Our study also indicates that the visualization and identification of the methylation profiles are essential for marker selection in future methylation studies.

It remains unclear whether specific mesenchymally methylated markers need to be developed. The majority of cells in most breast tumours are epithelial and thus should contribute to the majority of ctDNA. In addition, the majority of epithelially expressed (or mesenchymally methylated) markers are methylated at varying levels in peripheral blood DNA, which makes the major contribution to cell-free DNA and confounds the methylation-based detection of ctDNA. Thus, markers such as *RASSF1A*, *RARβ*, *TWIST1*, and *SFRP2*, whose methylation covers the epithelial–mesenchymal spectrum, are likely to be the most useful for monitoring ctDNA.

## 4. Materials and Methods

### 4.1. Breast Cancer Cell Lines

Frozen cell pellets of the following breast cancer cell lines, MDA-MB-231, BT-549, HCC1569, HCC1806, SK-BR-3, HCC70, MDA-MB-468, MDA-MB-453, MCF-7, HCC1954, HCC1419, T-47D, BT-20, BT-474, CAL-120, CAL-148, MCF 10A, SUM-149PT, SUM-159PT, UACC-732, UACC-893, ZR-75-1, MFM-223, and Hs578T were used to extract DNA for methylation analysis. These cell pellets were kindly provided by Riley Morrow and Tracy Cardwell from the Olivia-Newton John Cancer Research Institute, from cells that were originally from ATCC and/or had been Short Tandem Repeat (STR) authenticated. DNA from PMC42-ET and PMC42-LA cells was kindly provided by Mark Waltham, St. Vincent’s Hospital, Melbourne.

### 4.2. DNA Extraction from Cell Pellets

DNA was extracted from the pellets using the DNeasy^®^ Blood & Tissue Kit (Qiagen, Hilden, Germany) according to the manufacturer’s instructions. Briefly, in a screw cap tube (Neptune, San Diego, CA, USA), 200 µL of 1 × PBS and subsequently 20 µL of Proteinase K (Scimar, Cat no. LS004224, Lakewood, NJ, USA) were added to each cell pellet, which contained less than 5 × 10^6^ cells. The mixtures were vortexed briefly for 10 s. Then, 200 µL of Buffer AL was added into each cell pellet mixture. The mixtures were pulse-vortexed for 15 s and briefly centrifuged, followed by incubation in an oven at 56 °C overnight. The tubes containing cells and buffers were taken out of the oven and cooled for 5 min at room temperature before DNA extraction and clean-up. The tubes were vortexed and briefly centrifuged. Subsequently, 200 µL of ethanol (Sigma-Aldrich, Saint Louis, MO, USA) was added into each tube and the mixtures were pulse-vortexed and centrifuged. The contents in the screw cap tubes were carefully transferred into spin columns provided in the Blood & Tissue Kit, which were then centrifuged at 8000 rpm for 1 min. The flow through and collection tubes were discarded and new collection tubes were used for the subsequent washing steps. The spin columns were washed by adding 500 µL of Buffer AW1 and centrifuging at 8000 rpm for 1 min. Next, 500 µL of Buffer AW2 was added and the columns were centrifuged at 8000 rpm for 1 min. The spin columns were then centrifuged at 14,000 rpm for 3 min without adding any more washing buffer. During washing steps using the spin columns, the flow-through and collection tubes were discarded and the spin columns were inserted into new collection tubes for each step. To elute DNA, 50 µL of Buffer AE, which was prewarmed at 72 °C, was added directly into each spin column. After 5 min of incubation at room temperature, the columns were centrifuged at 14,000 rpm for 3 min. The DNA was then quantified using the DS-11 Spectrophotometer (DeNovix, Wilmington, DE, USA) and stored at 4 °C for short-term storage and at −20 °C for long-term storage.

### 4.3. Bisulfite Conversion

Bisulfite conversion was performed with the EZ DNA Methylation-Lightning kit (Zymo Research, Irvine, CA, USA). In total, 500–1000 ng of DNA was modified using the kit according to the manufacturer’s instructions. In 8-tube strips, PCR-grade water was added to the required volume of DNA to make up a total volume of 20 µL for each bisulfite converted reaction. Next, 130 µL of Lightning Conversion Reagent was added to each of the DNA solution, followed by pipetting up and down five times. After being centrifuged, the 8-tube strips were placed into a C1000 Touch™ thermal cycler (Bio-Rad, Hercules, CA, USA). The step-by-step cycling program was as follows: (1) denaturation step at 98 °C for 8 min, (2) incubation step at 54 °C for 60 min, and (3) hold at 4 °C for up to 20 h.

After the conversion, the bisulfite-converted DNA was cleaned up by transferring the DNA in the 8-tube strips to the Zymo-Spin™ IC columns containing 600 µL of M-binding buffer. The columns were inverted 5 times to mix the contents, and subsequently centrifuged at 14,000 rpm for 1 min. The flow-through was discarded and the spin columns were placed back into the collection tubes. Then, 100 µL of M-washing buffer was added to each spin column, which was centrifuged at 14,000 rpm for 1 min. Next, 200 µL of l-desulfonation buffer was added to each spin column, followed by a 20-min incubation at room temperature. The spin columns were centrifuged at 14,000 rpm for 1 min. The columns were washed by the addition of 200 µL of M-Wash buffer and centrifugation at 14,000 rpm for 1 min. This step was repeated once. After two washing steps, the spin columns were placed into new Eppendorf tubes. After adding 10 µL of M-Elution buffer directly into each spin column, the columns were incubated for 5 min at room temperature and then centrifuged at 14,000 rpm for 1 min. Another 10 µL of M-Elution buffer was added onto the spin columns, followed by 5-min incubation at room temperature and 14,000 rpm centrifugation for 1 min. A total of 20 µL of bisulfite converted DNA was obtained. Bisulfite converted DNA was stored at 4 °C for short-term storage and at −20 °C for long-term storage (longer than two months).

### 4.4. Methylation-Sensitive High-Resolution Melting

MS-HRM was performed on a Rotor-gene 6000 (Corbett, Sydney, Australia) or a CFX Connect Real-time System (BioRad, Hercules, CA, USA). For each MS-HRM run, a methylation standard series including 100%, 50%, 10%, and 3% methylated and fully unmethylated standards was included as a guide for detecting methylation and for calling methylation levels [23,25]. Each sample was run in duplicate. MS-HRM primers were designed following the guidelines described previously [22,63]. Reverse MS-HRM primers were biotinylated to allow the subsequent bisulfite pyrosequencing. The 20 µL of PCR reaction mix consisted of 1 × PCR buffer (Qiagen), 1.5 to 3.0 mM MgCl_2_, 200 µM dNTP mix (Fisher Biotech, Perth, Australia), 200–400 nM forward and reverse primers, 1 × SYTO9 intercalating dye (Thermo Fisher Scientific, Waltham, MA, USA), 0.5 U HotstarTaq polymerase (Qiagen), and 10 ng of bisulfite converted DNA (theoretical amount from the assumption of no DNA loss during bisulfite conversion). The primer sequences and the PCR conditions for all MS-HRM assays are listed in Tables S1 and S2 in Supplementary Materials, respectively.

The levels of homogeneous methylation were stated numerically as a percentage of methylation. The levels of heterogeneous methylation were described as ‘very high’, ‘high’, ‘moderate’, ‘low’, and ‘very low’ based on the extent to which the melting profiles extend to the methylated area.

### 4.5. Bisulfite Pyrosequencing

Bisulfite pyrosequencing was used subsequently to MS-HRM in order to quantify the methylation percentage at each CpG position [25]. The MS-HRM primers were used for bisulfite pyrosequencing. The reverse primers were biotinylated and forward primers were used as sequencing primers. Representative samples from *RASSF1A*, *RARβ*, *AKR1B1*, *GRHL2*, *CRABP1*, *SFRP2*, and *GFRA1* previous assessment by MS-HRM were pyrosequenced. Methylation standards were also included in pyrosequencing reactions. Two microlitres of PCR products from MS-HRM assays were used as templates for pyrosequencing.

The pyrosequencing reaction was performed on a Qseq pyrosequencer (Bio Molecular Systems, Sydney, Australia) using the Pyro Gold Q24 reagents (Qiagen). The injector set-up, priming, and testing were performed according to the Qseq instructions. After inserting the 48-well disc into the Qseq pyrosequencer, 8 µL of MilliQ water, 1 µL of well-mixed streptavidin Mag Sepharose beads, and 2 µL of PCR templates was loaded into each well. Denaturation of templates and subsequent annealing of biotinylated single-stranded DNA into beads were performed after the run was started. Two microlitres of the pyrosequencing primers (5 µM) were used for each reaction. Table S3 in Supplementary Materials provides the sequences before bisulfite treatment, sequences for analysis, and dispensation order for each assay. Qseq advanced software (Bio Molecular Systems, v2.0.11) was utilized to set up CpG assays (CpG mode) and run files. The obtained data were analysed and quantified with the Qseq Software. 

## 5. Conclusions

This is the first study to describe locus-specific DNA methylation status associated with the epithelial–mesenchymal spectrum. We determined the methylation status of a panel of genes in breast cancer cell lines that spanned the epithelial–mesenchymal spectrum. This included genes that might be expected to be epithelial or mesenchymal markers, in addition to established DNA methylation markers for monitoring MRD. We have shown that the methylation of many of these markers was indeed associated with the epithelial or mesenchymal status of the cell lines, whereas others were less specific. In addition, clustering using the methylation patterns generally supported the previously known gene expression-based classification of epithelial/mesenchymal status. Cancer markers previously used to detect MRD are predominantly methylated in epithelial and intermediate cell lines, with *RASSF1A*, *TWIST1*, and *RARβ* methylation covering more of the epithelial–mesenchymal spectrum in the cell line panel. A similar approach can be used to assess further markers in order to develop better DNA methylation marker panels for the detection of ctDNA in breast cancer.

## Figures and Tables

**Table 1:** Summary of hormonal receptor status, vimentin (VIM) status, molecular subtypes, and EMT status of the panel of 26 breast cancer cell lines according to the published literature. Breast cancer cell lines are ranked using the EMT score, from the most epithelial at the top, to the most mesenchymal at the bottom [11]. The EMT scores for PMC42-LA and PMC42-ET are not available. Hormonal receptor status, including estrogen receptor (ER), progesterone receptor (PR), and human epidermal growth factor receptor 2 (HER2) expression, were determined from (1) the American Type Culture Collection (ATCC) website and references therein [13], (2) Kao 2009 [14], and (3) Lehmann 2011 [15]. VIM status from immunofluorescent analysis was reported in (4) Thompson 1992 [17] and (5) Sommers 1994 [16]. The VIM status and the hormonal receptor status of PMC42-LA and PMC42-ET are cited from [12] and unpublished data. Molecular subtypes were obtained from the studies of Neve 2006 [18], Heiser 2012 [20] using the E-MTAB-181 dataset, and Hoeflich 2009 [19] using the GSE12790 dataset. The cell lines were also assigned the epithelial and mesenchymal status based on the study of Tan 2014 [11]. Unavailable information is left empty.

Ranking	BC Cell Lines	ER Expression	PR Expression	HER2 Expression	VIM Status	Subtypes	Subtypes	Subtypes	HER2 Status	EMT Status
Tan 2014		ATCC (1) Kao 2009 (2) Lehmann 2011 (3)			Thompson 1992 (4) Sommers 1994 (5)	Neve 2006	Heiser 2012 (E-MTAB-181)	Hoeflich 2009 (GSE12790)		Tan 2014
1	MFM-223	ER− (3)	PR− (3)	HER2− (3)				Luminal	Her2 amplified	Epithelial
2	MDA-MB-453	ER− (1)	PR− (1)	HER2− (1)	VIM− (5)	Luminal	Luminal	Luminal	Her2 amplified	Epithelial
3	BT-474	ER+ (2)	PR+ (2)	HER2+ (2)	VIM− (5)	Luminal	Luminal	Luminal	Her2 amplified	Epithelial
4	ZR-75-1	ER+ (2)	PR− (2)	HER2− (2)	VIM− (4)	Luminal	Luminal	Luminal	Nonamplified	Epithelial
5	HCC1419	ER− (1)	PR− (1)	HER2+ (1)			Luminal	Luminal	Her2 amplified	Epithelial
6	UACC-893	ER− (1)	PR− (1)	HER2+ (1)			Luminal	Luminal	Her2 amplified	Epithelial
7	SK-BR-3	ER− (2)	PR− (2)	HER2+ (2)	VIM− (4, 5)	Luminal	Luminal	Luminal	Her2 amplified	Epithelial
8	T-47D	ER+ (2)	PR+ (2)	HER− (2)	VIM− (4, 5)	Luminal	Luminal	Luminal	Nonamplified	Epithelial
9	CAL-148	ER− (3)	PR− (3)	HER2− (3)				Basal	Nonamplified	Epithelial
10	MCF-7	ER+ (2)	PR+ (2)	HER2− (2)	VIM− (4, 5)	Luminal	Luminal	Luminal	Nonamplified	Epithelial
11	UACC-732	ER− (1)	PR+ (1)	HER2+ (1)						Epithelial
12	MDA-MB-468	ER− (1)	PR− (1)	HER2− (1)	VIM− (4, 5)	Basal A	Basal A	Basal	Nonamplified	Intermediate
13	HCC1954	ER− (1)	PR− (1)	HER2+ (1)		Basal A	Basal A	Basal	Her2 amplified	Intermediate
14	HCC70	ER− (1)	PR− (1)	HER2− (1)		Basal A	Basal A	Basal	Nonamplified	Intermediate
15	BT-20	ER− (1)	PR− (1)	HER2− (1)		Basal A	Basal A	Basal	Nonamplified	Intermediate
16	HCC1806	ER− (1)	PR− (1)	HER2− (1)			Basal A	Basal	Nonamplified	Intermediate
17	HCC1569	ER− (1)	PR− (1)	HER2+ (1)		Basal A	Basal A	Basal	Her2 amplified	Intermediate
18	SUM-149PT	ER− (3)	PR− (3)	HER2− (3)		Basal B	Basal A			Intermediate
19	CAL-120	ER− (3)	PR− (3)	HER2− (3)				Basal	Nonamplified	Intermediate
20	MCF 10A	ER− (2)	PR− (2)	HER− (2)		Basal B	Basal A			Mesenchymal
	PMC42-LA	ER−	PR−	HER2−	VIM low					
	PMC42-ET	ER−	PR−	HER2−	VIM+					
21	MDA-MB-231	ER− (1)	PR− (1)	HER2− (1)	VIM+ (4, 5)	Basal B	Basal B	Basal	Nonamplified	Mesenchymal
22	BT-549	ER− (1)	PR− (1)	HER2− (1)	VIM+ (4, 5)	Basal B	Basal B	Basal	Nonamplified	Mesenchymal
23	SUM-159PT	ER− (3)	PR− (3)	HER2− (3)		Basal B	Basal B			Mesenchymal
24	Hs578T	ER− (1)	PR− (1)	HER2− (1)	VIM+ (4, 5)	Basal B	Basal B	Basal	Nonamplified	Mesenchymal

**Table 2:** Pyrosequencing results of representative samples for (**A**) *GRHL2* and (**B**) *RASSF1A*. Pyrosequencing data validated the results from MS-HRM and provided complementary information on methylation level at each CpG position.

A									
		% of Methylated Allele							
**CpG Positions** **Samples**	**Status by MS-HRM** **(Confirmed by Pyrosequencing)**	**1**	**2**	**3**	**4**	**5**	**6**	**7**	**8**
**100%M Std**		90	88	89	86	90	83	96	94
**50%M Std**		41	44	40	41	44	39	45	47
**10%M Std**		12	16	15	14	16	16	18	19
**0%M Std**		2	2	2	1	2	2	2	2
**BT-549**	high (yes)	67	88	79	96	93	89	83	70
**MCF 10A**	high (yes)	74	77	72	88	89	76	79	53
**MDA-MB-231**	moderate (yes)	17	27	15	25	20	23	21	13
**CAL-120**	very low (yes)	6	12	7	7	9	8	8	5
**HCC1806**	0%M (yes)	2	2	1	1	2	1	1	1
**B**									
		**% of Methylated Allele**							
**CpG Positions** **Samples**	**Status by MSHRM** **(Confirmed by Pyrosequencing)**	**1**		**2**		**3**		**4**	
**100%M Std**		97		65		95		89	
**50%M Std**		83		70		84		84	
**10%M Std**		39		26		44		40	
**0%M**		3		2		3		2	
**CAL-148**	100%M (yes)	89		92		96		96	
**SK-BR-3**	50%M (yes)	78		81		100		79	
**Hs578T**	10–50%M (yes)	72		64		71		71	
**MCF 10A**	moderate (yes)	55		37		52		41	
**HCC1806**	0%M (yes)	0		0		0		0	

90–10080–8950–7920–4910–190–9

**Table 3:** Methylation data of the studied markers on the panel of breast cancer cell lines. Cell lines were ranked in the epithelial–mesenchymal spectrum from the epithelial luminal (top), intermediate (middle), to mesenchymal basal (bottom) groups according to Tan et al. [11] unless indicated otherwise. The levels of homogeneous methylation were stated numerically as percentages of methylation. The estimated levels of heterogeneous methylation were described as ‘very high’, ‘high’, ‘moderate’, ‘low’, and ‘very low’. Zero means no methylation. ‘n.a.’ means no amplification. The methylation result is colour-coded to aid visualisation: red for 100% or very high methylation; orange for 50%, 50–100%, or high methylation; yellow for 10%, 10–50%, or moderate methylation; light yellow for 3%, 3–10%, or low methylation; no highlighting for 0%, very low methylation, or no amplification.

Breast Cancer Cell Lines	EMP-Unassigned Group							Mesenchymal Group					Epithelial Group		
	*AKR1B1*	*APC*	*RASSF1A*	*RARβ*	*SFRP2*	*GFRA1*	*CDKN2A*	*TWIST1*	*CRABP1*	*DKK3*	*EGFR*	*VIM*	*GRHL2*	*MIR200C*	*CDH1*
**MFM-223**	very high	100%	100%	100%	10–50%	100%	moderate	high	100%	100%	low	very low	0	0	0
**MDA-MB-453**	very high	100%	moderate	100%	50–100%	high	0	high	moderate	100%	10–50%	100%	0	0	0
**BT-474**	100%	10–50%	100%	100%	50–100%	0	0	very low	high	moderate	very low	0	0	0	0
**ZR-75-1**	moderate	100%	100%	high	moderate	very low	low	moderate	low	low	very low	moderate	0	0	0
**HCC1419**	very high	0	100%	very low	high	0	0	very low	high	moderate	very low	moderate	0	0	0
**UACC-893**	very high	50%	100%	high	high	moderate	0	high	low	moderate	0	low	0	0	0
**SK-BR-3**	moderate	low	50%	high	moderate	0	0	very low	0	0	very low	moderate	0	0	n.a.
**T-47D**	moderate	0	100%	very low	moderate	0	100%	high	moderate	moderate	moderate	100%	0	0	0
**CAL-148**	very high	100%	100%	high	moderate	0	0	100%	high	low	very low	moderate	0	0	0
**MCF-7**	very high	10–50%	100%	100%	high	0	n.a.	high	moderate	moderate	low	100%	0	0	0
**UACC-732**	high	50%	100%	high	moderate	0	0	high	low	moderate	very high	100%	0	0	0
**MDA-MB-468**	0	3%	100%	50–100%	moderate	moderate	0	high	low	moderate	0	0	0	0	0
**HCC1954**	100%	100%	100%	high	high	low	100%	moderate	moderate	high	very low	0	0	0	0
**HCC70**	0	0	0	50%	very low	low	0	moderate	0	moderate	very low	0	0	0	0
**BT-20**	0	100%	50%	100%	100%	100%	n.a.	100%	high	100%	0	100%	0	0	0
**HCC1806**	0	0	0	very low	0	0	n.a.	0	very low	0	very low	very low	0	0	0
**HCC1569**	10–50%	50–100%	10–50%	very low	100%	100%	low	100%	0	moderate	very low	0	0	0	0
**SUM-149PT**	0	0	0	moderate	low	0	n.a.	very low	very low	very low	very low	0	0	0	0
**CAL-120**	0	0	0	50%	moderate	0	0	very low	low	0	very low	0	very low	very low	very low
**MCF 10A**	0	0	moderate	high	low	low	n.a.	very low	very low	low	0	0	high	0	0
**PMC42-LA**	0	0	moderate	100%	low	0	n.a.	low	very low	0	0	0	10%	low	0
**PMC42-ET**	0	0	moderate	50–100%	moderate	0	n.a.	moderate	low	0	0	0	50–100%	high	very low
**MDA-MB-231**	0	0	50%	very low	high	moderate	n.a.	100%	low	high	very low	10%	moderate	moderate	moderate
**BT-549**	0	0	very low	very low	0	0	0	low	low	very low	very low	0	high	moderate	very low
**SUM-159PT**	0	0	50%	moderate	high	low	very low	high	0	0	very low	0	high	high	moderate
**Hs578T**	0	0	10–50%	0	very low	n.a.	n.a.	high	0	0	0	0	moderate	high	moderate

## Supplementary Materials

Supplementary materials can be found at http://www.mdpi.com/1422-0067/19/9/2553/s1.

## Abbreviations

EMP	Epithelial–mesenchymal plasticity
EMT	Epithelial–mesenchymal transition
MET	Mesenchymal–epithelial transition
MS-HRM	Methylation-sensitive high-resolution melting
PCR	Polymerase chain reaction
MSP	Methylation-specific PCR
CpG	Cytosine–guanine dinucleotides
MRD	Minimal residual disease
CTCs	Circulating tumour cells
ctDNA	Circulating tumour DNA
ER	Estrogen receptor
PR	Progesterone receptor
HER2	Human epidermal growth factor receptor 2
ATCC	American Type Culture Collection
AKR1B1	Aldo-Keto Reductase Family 1 Member B1
APC	Adenomatous Polyposis Coli
RASSF1A	Ras Association Domain Family Member 1A
RAR*ß*	Retinoic Acid Receptor Beta
CDKN2A	Cyclin-Dependent Kinase Inhibitor 2A
BRCA1	Breast Cancer 1
TWIST1	Twist Family BHLH Transcription Factor 1
CRABP1	Cellular Retinoic Acid Binding Protein 1
DKK3	Dickkopf WNT Signaling Pathway Inhibitor 3
EGFR	Epidermal Growth Factor Receptor
VIM	Vimentin
GRHL2	Grainyhead-Like Transcription Factor 2
MIR200C	MicroRNA 200c
CDH1	Cadherin 1
SFRP2	Secreted Frizzled Related Protein 2
GFRA1	GDNF Family Receptor Alpha 1

## References

[1] Silva J.M., Dominguez G., Garcia J.M., Gonzalez R., Villanueva M.J., Navarro F., Bonilla F. (1999). Presence of tumor DNA in plasma of breast cancer patients: Clinicopathological correlations. Cancer Res..

[2] Cancer Genome Atlas Network (2012). Comprehensive molecular portraits of human breast tumours. Nature.

[3] Greenburg G., Hay E.D. (1982). Epithelia suspended in collagen gels can lose polarity and express characteristics of migrating mesenchymal cells. J. Cell Biol..

[4] Liu F., Zhou Y., Zhou D., Kan M., Niu X., Zhang Z., Liu Y. (2014). Whole DNA methylome profiling in lung cancer cells before and after epithelial-to-mesenchymal transition. Diagn. Pathol..

[5] Cardenas H., Vieth E., Lee J., Segar M., Liu Y., Nephew K.P., Matei D. (2014). TGF-beta induces global changes in DNA methylation during the epithelial-to-mesenchymal transition in ovarian cancer cells. Epigenetics.

[6] Cardenas H., Vieth E., Lee J., Segar M., Liu Y., Nephew K.P., Matei D. (2010). Genome-wide analysis of aberrant methylation in human breast cancer cells using methyl-DNA immunoprecipitation combined with high-throughput sequencing. BMC Genom..

[7] Carmona F.J., Davalos V., Vidal E., Gomez A., Heyn H., Hashimoto Y., Sánchez-Mut J.V. (2014). A comprehensive DNA methylation profile of epithelial-to-mesenchymal transition. Cancer Res..

[8] Thiery J.P. (2002). Epithelial-mesenchymal transitions in tumour progression. Nat. Rev. Cancer.

[9] Chaffer C.L., San Juan B.P., Lim E., Weinberg R.A. (2016). EMT, cell plasticity and metastasis. Cancer Metast. Rev..

[10] Dongre A., Rashidian M., Reinhardt F., Bagnato A., Keckesova Z., Ploegh H.L., Weinberg R.A. (2017). Epithelial-to-Mesenchymal Transition Contributes to Immunosuppression in Breast Carcinomas. Cancer Res..

[11] Tan T.Z., Miow Q.H., Miki Y., Noda T., Mori S., Huang R.Y.J., Thiery J.P. (2014). Epithelial-mesenchymal transition spectrum quantification and its efficacy in deciphering survival and drug responses of cancer patients. EMBO Mol. Med..

[12] Blick T., Widodo E., Hugo H., Waltham M., Lenburg M.E., Neve R.M., Thompson E.W. (2008). Epithelial mesenchymal transition traits in human breast cancer cell lines. Clin. Exp. Metast..

[13] America Type Culture Collection. https://www.atcc.org.

[14] Kao J., Salari K., Bocanegra M., Choi Y.L., Girard L., Gandhi J., Minna J.D. (2009). Molecular profiling of breast cancer cell lines defines relevant tumor models and provides a resource for cancer gene discovery. PLoS ONE.

[15] Lehmann B.D., Bauer J.A., Chen X., Sanders M.E., Chakravarthy A.B., Shyr Y., Pietenpol J.A. (2011). Identification of human triple-negative breast cancer subtypes and preclinical models for selection of targeted therapies. J. Clin. Investig..

[16] Sommers C.L., Byers S.W., Thompson E.W., Torri J.A., Gelmann E.P. (1994). Differentiation state and invasiveness of human breast cancer cell lines. Breast Cancer Res. Treat..

[17] Thompson E.W., Paik S., Brünner N., Sommers C.L., Zugmaier G., Clarke R., Martin G.R. (1992). Association of increased basement membrane invasiveness with absence of estrogen receptor and expression of vimentin in human breast cancer cell lines. J. Cell. Physiol..

[18] Neves P.L., Morgado E., Faísca M., Carrasqueira H., Baptista A., Silva A.P. (2006). Nutritional and inflammatory status influence darbepoetin dose in pre-dialysis elderly patients. Int. Urol. Nephrol..

[19] Hoeflich K.P., O’Brien C., Boyd Z., Cavet G., Guerrero S., Jung K., Zhou W. (2009). In vivo antitumor activity of MEK and phosphatidylinositol 3-kinase inhibitors in basal-like breast cancer models. Clin. Cancer Res..

[20] Heiser L.M., Sadanandam A., Kuo W.L., Benz S.C., Goldstein T.C., Ng S., Bayani N. (2012). Subtype and pathway specific responses to anticancer compounds in breast cancer. Proc. Natl. Acad. Sci. USA.

[21] Kalous O., Conklin D., Desai A.J., O’Brien N.A., Ginther C., Anderson L., Slamon D.J. (2012). Dacomitinib (PF-00299804), an irreversible Pan-HER inhibitor, inhibits proliferation of HER2-amplified breast cancer cell lines resistant to trastuzumab and lapatinib. Mol. Cancer Ther..

[22] Wojdacz T.K., Dobrovic A., Hansen L.L. (2008). Methylation-sensitive high-resolution melting. Nat. Protoc..

[23] Wojdacz T.K., Dobrovic A. (2007). Methylation-sensitive high resolution melting (MS-HRM): A new approach for sensitive and high-throughput assessment of methylation. Nucleic Acids Res..

[24] Candiloro I.L., Mikeska T., Hokland P., Dobrovic A. (2008). Rapid analysis of heterogeneously methylated DNA using digital methylation-sensitive high resolution melting: Application to the CDKN2B (p15) gene. Epigenet. Chromatin.

[25] Candiloro I.L., Mikeska T., Dobrovic A. (2011). Assessing combined methylation-sensitive high resolution melting and pyrosequencing for the analysis of heterogeneous DNA methylation. Epigenetics.

[26] Veeck J., Bektas N., Hartmann A., Kristiansen G., Heindrichs U., Knüchel R., Dahl E. (2008). Wnt signalling in human breast cancer: Expression of the putative Wnt inhibitor Dickkopf-3 (DKK3) is frequently suppressed by promoter hypermethylation in mammary tumours. Breast Cancer Res..

[27] Montero A.J., Díaz-Montero C.M., Mao L., Youssef E., Estecio M.R., Shen L., Issa J.P. (2006). Epigenetic inactivation of EGFR by CpG island hypermethylation in cancer. Cancer Biol. Ther..

[28] Paz M.F., Fraga M.F., Avila S., Guo M., Pollan M., Herman J.G., Esteller M. (2003). A systematic profile of DNA methylation in human cancer cell lines. Cancer Res..

[29] Lombaerts M., Van Wezel T., Philippo K., Dierssen J.W.F., Zimmerman R.M.E., Oosting J., Cleton-Jansen A.M. (2006). E-cadherin transcriptional downregulation by promoter methylation but not mutation is related to epithelial-to-mesenchymal transition in breast cancer cell lines. Br. J. Cancer.

[30] Cieply B., Farris J., Denvir J., Ford H.L., Frisch S.M. (2013). Epithelial-mesenchymal transition and tumor suppression are controlled by a reciprocal feedback loop between ZEB1 and Grainyhead-like-2. Cancer Res..

[31] Xiang X., Deng Z., Zhuang X., Ju S., Mu J., Jiang H., Zhang H.G. (2012). Grhl2 determines the epithelial phenotype of breast cancers and promotes tumor progression. PLoS ONE.

[32] Cieply B., Riley P., Pifer P.M., Widmeyer J., Addison J.B., Ivanov A.V., Frisch S.M. (2012). Suppression of the epithelial-mesenchymal transition by Grainyhead-like-2. Cancer Res..

[33] Walter K., Holcomb T., Januario T., Du P., Evangelista M., Kartha N., Modrusan Z. (2012). DNA methylation profiling defines clinically relevant biological subsets of non-small cell lung cancer. Clin. Cancer Res..

[34] Davalos V., Moutinho C., Villanueva A., Boque R., Silva P., Carneiro F., Esteller M. (2012). Dynamic epigenetic regulation of the microRNA-200 family mediates epithelial and mesenchymal transitions in human tumorigenesis. Oncogene.

[35] Neves R., Scheel C., Weinhold S., Honisch E., Iwaniuk K.M., Trompeter H.I., Uhrberg M. (2010). Role of DNA methylation in miR-200c/141 cluster silencing in invasive breast cancer cells. BMC Res. Notes.

[36] Lim Y., Wright J.A., Attema J.L., Gregory P.A., Bert A.G., Smith E., Goodall G.J. (2013). Epigenetic modulation of the miR-200 family is associated with transition to a breast cancer stem-cell-like state. J. Cell Sci..

[37] Katz E., Dubois-Marshall S., Sims A.H., Faratian D., Li J., Smith E.S., Langdon S.P. (2010). A gene on the HER2 amplicon, C35, is an oncogene in breast cancer whose actions are prevented by inhibition of Syk. Br. J. Cancer.

[38] Vlahov N., Scrace S., Soto M.S., Grawenda A.M., Bradley L., Pankova D., Timpson P. (2015). Alternate RASSF1 Transcripts Control SRC Activity, E-Cadherin Contacts, and YAP-Mediated Invasion. Curr. Biol..

[39] Donninger H., Vos M.D., Clark G.J. (2007). The RASSF1A tumor suppressor. J. Cell Sci..

[40] Shinozaki M., Hoon D.S., Giuliano A.E., Hansen N.M., Wang H.J., Turner R., Taback B. (2005). Distinct hypermethylation profile of primary breast cancer is associated with sentinel lymph node metastasis. Clin. Cancer Res..

[41] Gort E.H., Suijkerbuijk K.P., Roothaan S.M., Raman V., Vooijs M., van der Wall E., van Diest P.J. (2008). Methylation of the TWIST1 promoter, TWIST1 mRNA levels, and immunohistochemical expression of TWIST1 in breast cancer. Cancer Epidemiol. Biomarkers Prev..

[42] Veeck J., Noetzel E., Bektas N., Jost E., Hartmann A., Knüchel R., Dahl E. (2008). Promoter hypermethylation of the SFRP2 gene is a high-frequent alteration and tumor-specific epigenetic marker in human breast cancer. Mol. Cancer.

[43] Zhang X.I.E., Zhang X., Sun B., Lu H., Wang D., Yuan X., Huang Z. (2014). Detection of aberrant promoter methylation of RNF180, DAPK1 and SFRP2 in plasma DNA of patients with gastric cancer. Oncol. Lett..

[44] Kohno H., Amatya V.J., Takeshima Y., Kushitani K., Hattori N., Kohno N., Inai K. (2010). Aberrant promoter methylation of WIF-1 and SFRP1, 2, 4 genes in mesothelioma. Oncol. Rep..

[45] Nojima M., Suzuki H., Toyota M., Watanabe Y., Maruyama R., Sasaki S., Hirata K. (2007). Frequent epigenetic inactivation of SFRP genes and constitutive activation of Wnt signaling in gastric cancer. Oncogene.

[46] Suzuki H., Gabrielson E., Chen W., Anbazhagan R., Van Engeland M., Weijenberg M.P., Baylin S.B. (2002). A genomic screen for genes upregulated by demethylation and histone deacetylase inhibition in human colorectal cancer. Nat. Genet..

[47] Sun Y., Zhu D., Chen F., Qian M., Wei H., Chen W., Xu J. (2016). SFRP2 augments WNT16B signaling to promote therapeutic resistance in the damaged tumor microenvironment. Oncogene.

[48] Klaus A., Birchmeier W. (2008). Wnt signalling and its impact on development and cancer. Nat. Rev. Cancer.

[49] Jönsson M., Borg Å., Nilbert M., Andersson T. (2000). Involvement of adenomatous polyposis coli (APC)/beta-catenin signalling in human breast cancer. Eur. J. Cancer.

[50] Fearon E.R., Vogelstein B. (1990). A genetic model for colorectal tumorigenesis. Cell.

[51] Heuberger J., Birchmeier W. (2010). Interplay of cadherin-mediated cell adhesion and canonical Wnt signaling. Cold Spring Harb. Perspect. Biol..

[52] Zablocki G.J., Ruzycki P.A., Overturf M.A., Palla S., Reddy G.B., Petrash J.M. (2011). Aldose reductase-mediated induction of epithelium-to-mesenchymal transition (EMT) in lens. Chem. Biol. Interact..

[53] Wu X., Li X., Fu Q., Cao Q., Chen X., Wang M., Wang D. (2017). AKR1B1 promotes basal-like breast cancer progression by a positive feedback loop that activates the EMT program. J. Exp. Med..

[54] Dedeurwaerder S., Desmedt C., Calonne E., Singhal S.K., Haibe-Kains B., Defrance M., Lallemand F. (2011). DNA methylation profiling reveals a predominant immune component in breast cancers. EMBO Mol. Med..

[55] Fackler M.J., Umbricht C., Williams D., Argani P., Cruz L.A., Merino V.F., Marks J. (2011). Genome-wide methylation analysis identifies genes specific to breast cancer hormone receptor status and risk of recurrence. Cancer Res..

[56] Lee J.S., Fackler M.J., Lee J.H., Choi C., Park M.H., Yoon J.H., Sukumar S. (2010). Basal-like breast cancer displays distinct patterns of promoter methylation. Cancer Biol. Ther..

[57] Su Y., Subedee A., Bloushtain-Qimron N., Savova V., Krzystanek M., Li L., Li M. (2015). Somatic Cell. Fusions Reveal Extensive Heterogeneity in Basal-like Breast Cancer. Cell Rep..

[58] Chung V.Y., Tan T.Z., Tan M., Wong M.K., Kuay K.T., Yang Z., Thiery J.P. (2016). GRHL2-miR-200-ZEB1 maintains the epithelial status of ovarian cancer through transcriptional regulation and histone modification. Sci. Rep..

[59] Malouf G.G., Taube J.H., Lu Y., Roysarkar T., Panjarian S., Estecio M.R., Tahara T. (2013). Architecture of epigenetic reprogramming following Twist1-mediated epithelial-mesenchymal transition. Genome Biol..

[60] Cope L.M., Fackler M.J., Lopez-Bujanda Z., Wolff A.C., Visvanathan K., Gray J.W., Umbricht C.B. (2014). Do breast cancer cell lines provide a relevant model of the patient tumor methylome?. PLoS ONE.

[61] Smiraglia D.J., Rush L.J., Frühwald M.C., Dai Z., Held W.A., Costello J.F., Caligiuri M.A. (2001). Excessive CpG island hypermethylation in cancer cell lines versus primary human malignancies. Hum. Mol. Genet..

[62] Mikeska T., Bock C., Do H., Dobrovic A. (2012). DNA methylation biomarkers in cancer: Progress towards clinical implementation. Expert Rev. Mol. Diagn..

[63] Wojdacz T.K., Hansen L.L., Dobrovic A. (2008). A new approach to primer design for the control of PCR bias in methylation studies. BMC Res. Notes.

