# Supplementary material for: DNA Methylation Profiling of Breast Cancer Cell Lines along the Epithelial Mesenchymal Spectrum—Implications for the Choice of Circulating Tumour DNA Methylation Markers
Source: Int J Mol Sci. 2018 Aug 28;19(9):2553. doi: 10.3390/ijms19092553 (PMC6164039; doi:10.3390/ijms19092553)
Supplement: Supplementary file 1 [file ijms-19-02553-s001.zip › Additional file 1_Sup fig1.pdf]

A

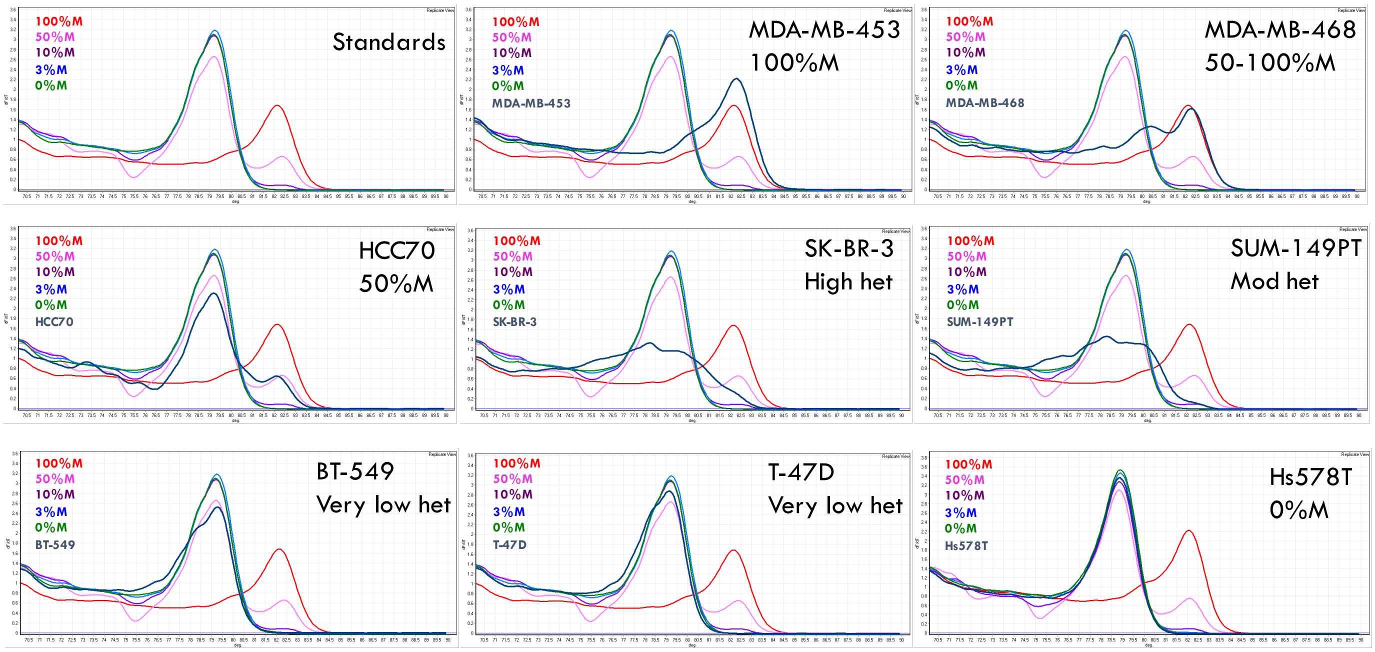

B

CpG positions / Samples	Status by MSHRM (confirmed by Pyrosequencing)	% of methylated allele 1	2	3	4
100%M Std		94	96	96	96
50%M Std		31	33	34	33
10%M Std		9	9	8	9
0%M Std		1	1	1	2
MDA-MB-453	100%M ✔	97	96	97	98
MDA-MB-468	50-100%M ✔	84	89	90	96
HCC70	50%M ✔	48	50	48	50
SK-BR-3	high het ✔	65	50	50	78
SUM-149PT	mod het ✔	32	28	28	85
BT-549	very low het ✔	7	5	6	5
T-47D	very low het ✔	2	2	3	4
Hs578T	0%M ✔	2	2	2	2

90-100
80-89
50-79
20-49
10-19
0-9

C

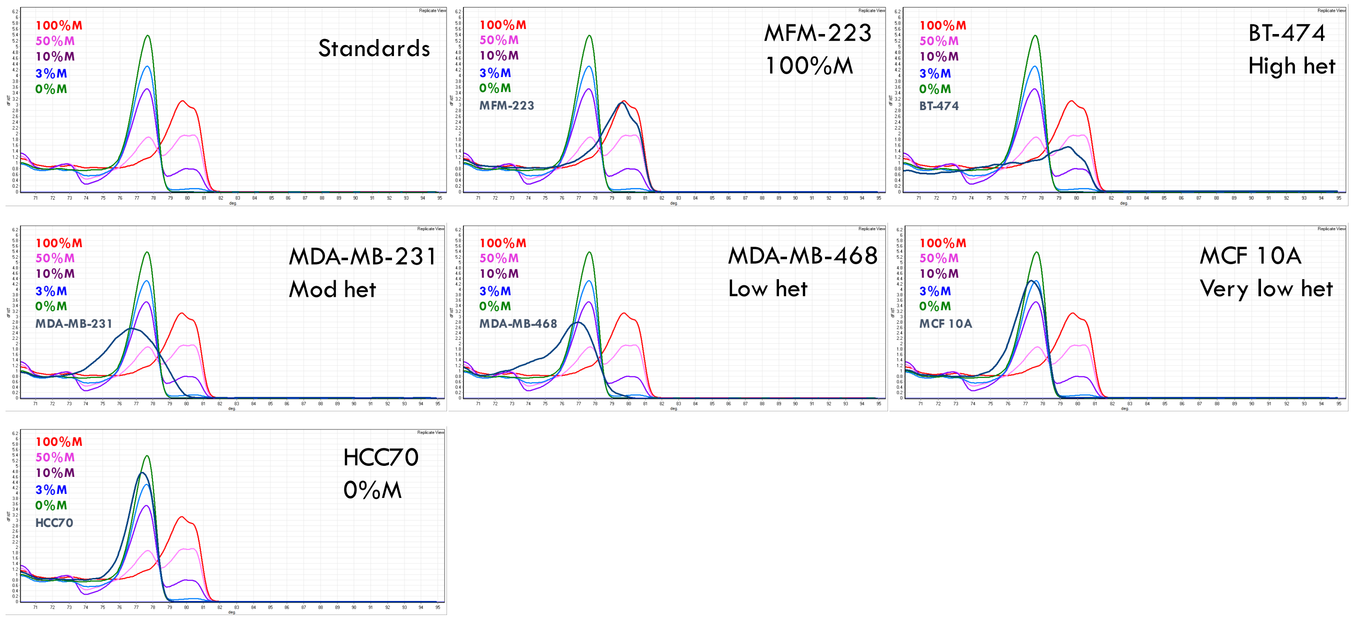

D

CpG positions / Samples	Status by MSHRM (confirmed by Pyrosequencing)	% of methylated allele 1	2	3	4	5	6	7	8	9	10
100%M Std		93	90	83	95	93	81	97	96	91	86
50%M Std		76	82	71	78	74	74	75	72	81	78
10%M Std		35	37	36	38	39	35	36	41	39	32
0%M Std		4	3	5	4	3	0	3	0	4	0
MFM-223	100%M ✔	92	93	84	90	86	86	87	99	96	88
BT-474	high het ✔	83	74	72	78	84	63	78	84	77	53
MDA-MB-231	mod het ✔	89	58	75	18	15	6	18	46	37	64
MDA-MB-468	low het ✔	26	23	18	17	14	7	15	36	22	46
MCF 10A	very low het ✔	5	3	7	2	2	0	3	7	6	5
HCC70	0%M ✔	3	7	6	2	6	3	0	12	4	5

90-100
80-89
50-79
20-49
10-19
0-9

E

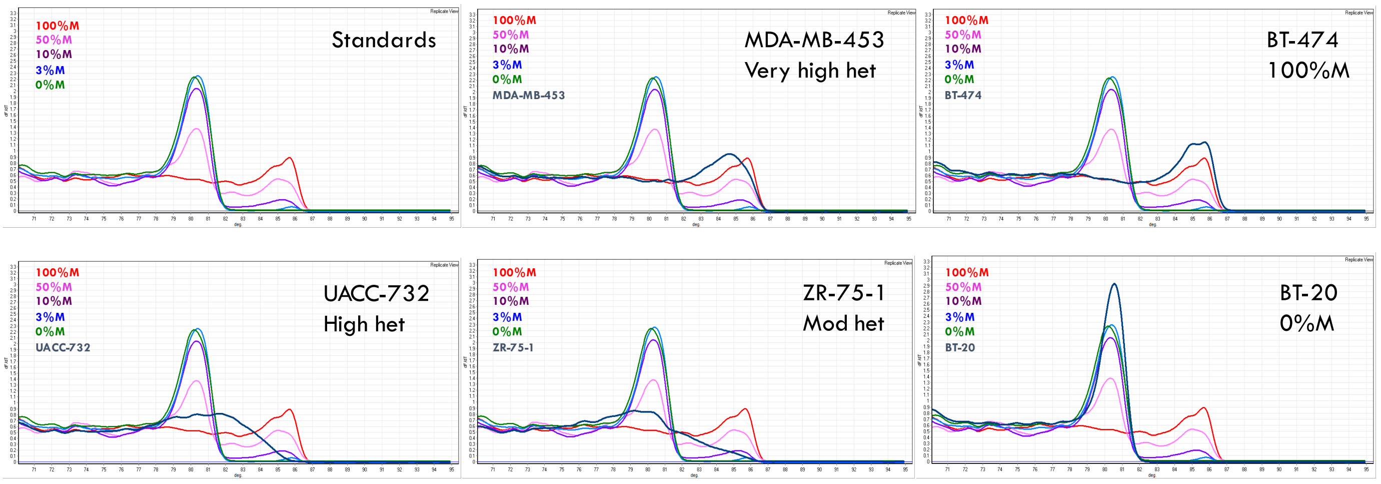

F

		% of methylated allele											
CpG positions Samples	Status by MSHRM (confirmed by Pyrosequencing)	1	2	3	4	5	6	7	8	9	10	11	12
100%M Std		84	82	89	80	89	84	81	84	78	83	78	79
50%M Std		50	45	47	47	50	50	45	45	41	44	42	46
10%M Std		19	18	19	19	16	19	10	19	17	14	17	20
0%M Std		6	0	2	2	2	0	0	0	0	3	0	13
MDA-MB-453	very high het ✔	95	93	96	97	95	94	91	91	84	95	85	91
BT-474	100%M ✔	96	95	96	97	96	95	97	100	90	95	83	89
UACC-732	high het ✔	97	89	93	90	94	96	28	44	33	60	55	64
ZR-75-1	mod het ✔	68	41	42	50	55	61	66	34	48	65	59	46
BT-20	0%M ✔	2	2	2	2	1	1	2	2	2	1	0	2

90-100
80-89
50-79
20-49
10-19
0-9

G

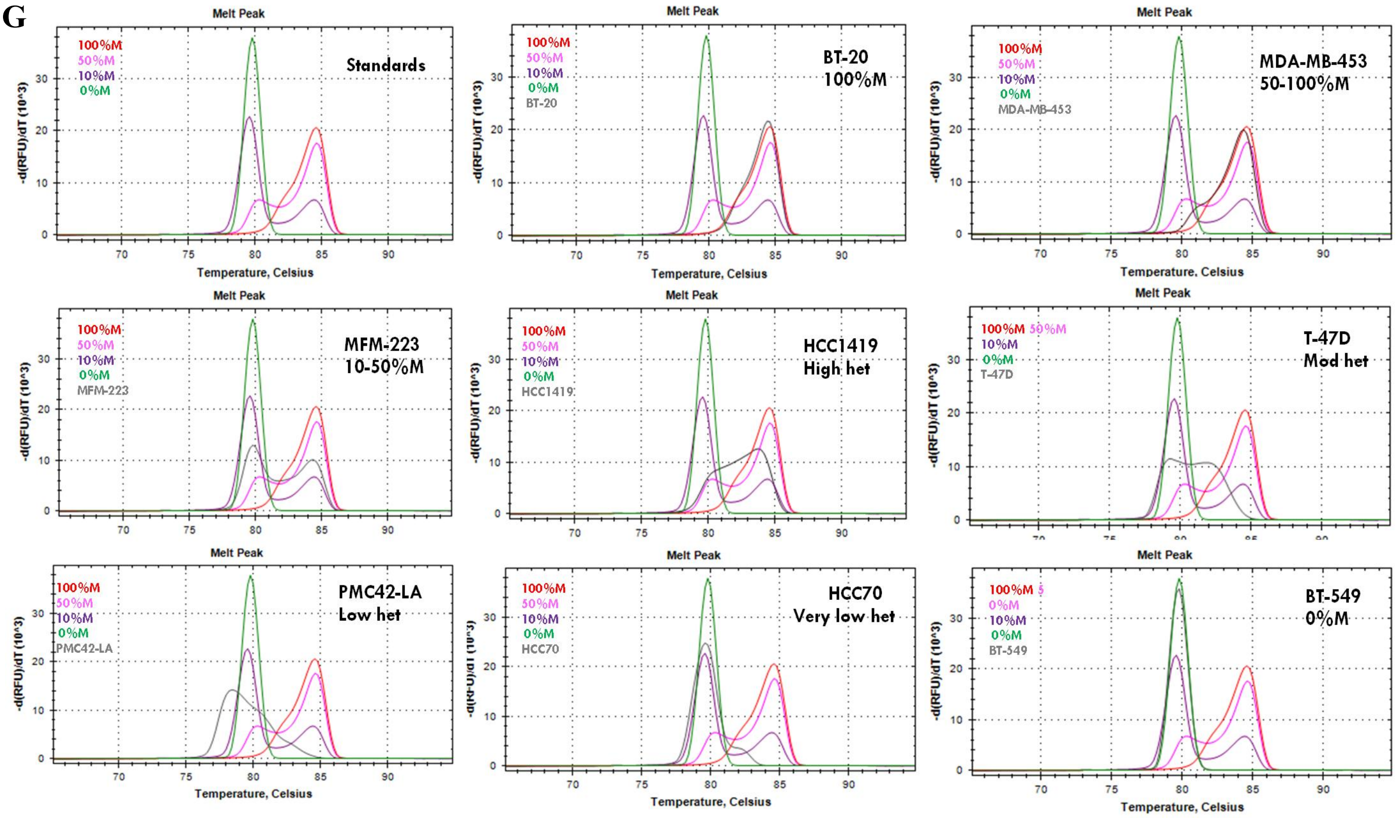

H

CpG positions Samples	Status by MSHRM (confirmed by Pyrosequencing)	% of methylated allele 1	2	3	4	5	6	7	8	9	10	11	12	13	14	15	16	17
100%M Std		85	97	94	100	92	81	89	84	100	100	79	97	100	100	64	93	87
50%M Std		71	74	74	79	72	69	68	66	79	80	63	88	81	87	48	70	65
10%M Std		27	36	29	34	32	28	28	23	34	30	23	32	33	38	20	23	31
0%M Std		3	7	4	3	3	0	0	3	5	4	0	4	4	0	0	4	3
BT-20	100%M ✔	73	70	90	100	95	88	89	85	94	100	80	96	96	100	68	83	78
MDA-MB-453	50-100%M✔	69	55	87	94	94	84	89	82	98	100	85	100	100	100	74	93	82
MFM-223	10-50%M✔	47	54	48	54	52	48	45	42	49	57	43	51	66	97	43	55	51
HCC1419	High het✔	30	24	75	82	55	60	65	77	77	96	69	98	97	97	70	98	89
T-47D	Mod het✔	58	7	22	34	29	25	28	52	43	66	49	37	74	100	55	81	84
PMC42-LA	low het✔	9	7	8	16	19	14	18	38	33	47	24	37	54	78	30	35	31
HCC70	very low het✔	7	8	6	4	10	3	3	22	12	27	14	12	14	24	15	24	22
BT-549	0%M✔	0	5	3	6	0	3	4	7	3	0	3	3	0	0	0	4	0

Legend
90-100
80-89
50-79
20-49
10-19
0-9

I

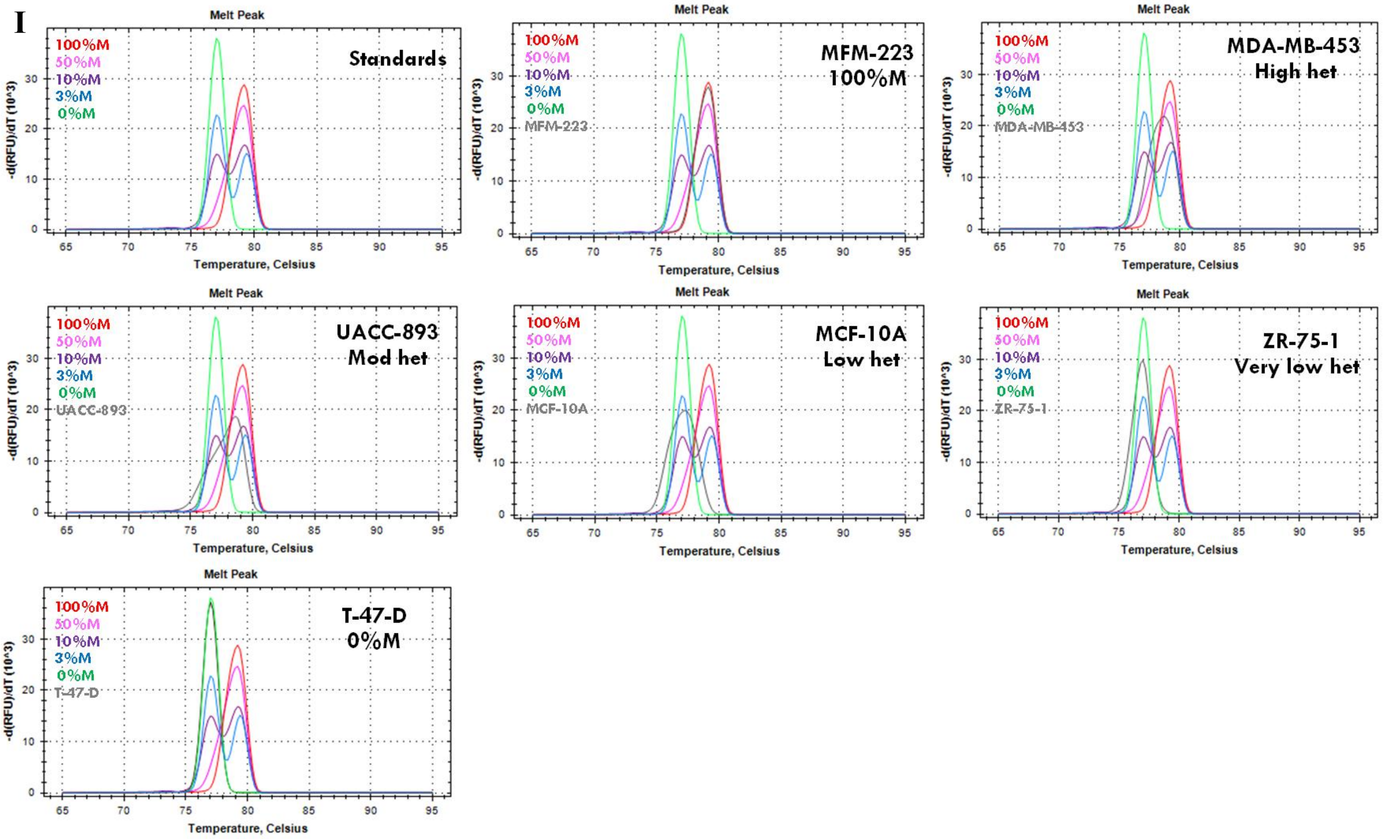

J

CpG positions Samples	Status by MSHRM (confirmed by Pyrosequencing)	% of methylated allele 1	2	3	4	5	6	7	8
100%M Std		100	98	89	89	74	88	85	75
50%M Std		96	95	85	88	78	91	83	77
10%M Std		59	55	52	53	44	51	45	42
3%M Std		38	36	33	35	27	34	32	30
0%M Std		1	2	0	1	0	4	5	0
MFM-223	100%M ✔	97	99	89	91	66	92	88	79
MDA-MB-453	high het ✔	75	79	88	91	62	80	85	75
UACC-893	mod het ✔	30	36	80	78	53	87	78	69
MCF 10A	low het ✔	7	12	24	54	31	30	59	51
ZR-75-1	very low het ✔	5	2	6	15	3	17	15	6
T-47D	0%M ✔	0	2	12	5	1	2	2	2

Legend
90-100
80-89
50-79
20-49
10-19
0-9
