# Supplementary material for: DNA Methylation Profiling of Breast Cancer Cell Lines along the Epithelial Mesenchymal Spectrum—Implications for the Choice of Circulating Tumour DNA Methylation Markers
Source: Int J Mol Sci. 2018 Aug 28;19(9):2553. doi: 10.3390/ijms19092553 (PMC6164039; doi:10.3390/ijms19092553)
Supplement: Supplementary file 1 [file ijms-19-02553-s001.zip › Additional file 2_Table S1.docx]

**Table S1: Oligonucleotide sequences for MS-HRM assays and characteristics of the corresponding amplicons**

| Gene | Primer sequences  (CpG sites in bold, converted Cs as capital Ts or As) | Strands | Amplicon size (bp) | No. of CpGs between primers | Spanned regions (UCSC Genome Browser on Human Feb. 2009 (GRCh37/hg19) Assembly) |
| --- | --- | --- | --- | --- | --- |
| *APC* | Forward: **cg**gggTTTtgtgTTTTaTtg  Reverse: tccaA**cg**AattacacaActAc | Forward | 71 | 4 | chr5:112,073,406-112,073,476 |
| *RASSF1A* | Forward: t**cg**ggTTTTaTagtTTTtgTaTTTaggtttT  Reverse: cctcccccaAAatccaAactAA | Reverse | 87 | 4 | chr3:50,378,323-50,378,409 |
| *RARβ* | Forward: **cg**agTtgtttgaggaTtgggatgT  Reverse: a**cg**AtAcccaAacaaaccctAct**c** | Forward | 66 | 5 | chr3:25,469,836-25,469,901 |
| *AKR1B1* | Forward: **g**gTaTTTTTag**cg**TaaTTaatTagaaggTt  Reverse: aAcaAAaAacgActtAccatAActActAc | Reverse | 172 | 17 | chr7:134,143,795-134,143,966 |
| *CDKN2A* | Forward: TTT**cg**ggggagaTTTaaTTtgg  Reverse: **cg**accctAtccctcaaatcctctA | Reverse | 152 | 9 | chr9:21,974,958-21,975,109 |
| *BRCA1* | Forward: TtgTtgTttag**cg**gtagTTTTttggtt  Reverse: caAt**cg**caAttttaatttatctAtaattcc**c** | Reverse | 79 | 4 | chr17:41,277,396-41,277,474 |
| *SFRP2* | Forward: aaaaggTTaagaaaaTtTtggTtgtgTTTTag  Reverse: ct**cg**aaaccc**cg**aaaaActAAcaAc | Forward | 170 | 19 | chr10:116,275,122-116,275,272 |
| *GFRA1* | Forward: gTt**cg**ggatTtttgTtgatgtTTaagttTatT  Reverse: **cg**atcaaaatAaAccattcttAtctAtcct | Forward | 150 | 10 | chr4:153,788,939-153,789,108 |
| *GRHL2* | Forward: **cg**TTaTtttTtgTtTtgtgtTtgTTTattgTTa  Reverse: tAAAataaAAAAAAAcccctcacatAAAAcc | Forward | 107 | 8 | chr8:102,504,695-102,504,801 |
| *MIR200C* | Forward: **cg**agtTTTtggggaTaTttTTtggtga  Reverse: AActAc**cg**AAAAtaAAAAaaAAtAActcaAaAA**c** | Reverse | 161 | 9 | chr12:7,072,606-7,072,766 |
| *CDH1* | Forward: gagTttg**cg**gaagtTagttTagaTtTTag  Reverse: cgActccaaAAAcccatAActAAc**c** | Forward | 113 | 11 | chr16:68,771,223-68,771,335 |
| *CRABP1* | Forward: **gcg**TTttgagtTaTtaggagag  Reverse: **cg**cacctctAAaAActAaAAcac | Reverse | 143 | 10 | chr15:78,632,530-78,632,672 |
| *TWIST1* | Forward: tgtTaTagTTaTtT**cg**gatggggTtgT  Reverse: **cg**AAA**cg**atttccttccc**c** | Reverse | 124 | 11 | chr7:19,157,542-19,157,665 |
| *DKK3* | Forward: **cg**aTtTtgTtgagTtTagTTtTtTttggtgg  Reverse: AcacaAAtcaAccccctcccct | Reverse | 89 | 5 | chr11:12,030,600-12,030,688 |
| *VIM* | Forward: t**cg**TTaTTTtT**cg**TagTTatgtTTaTTaggt  Reverse: caA**cgcg**ctAcccaAActAtaAAtA**c** | Forward | 150 | 14 | chr10:17,271,404-17,271,553 |
| *EGFR* | Forward: **g**gag**cg**agTtTtt**cg**gggagTag  Reverse: **g**cccttacctttcttttcctccaA | Forward | 121 | 11 | chr7:55,086,946-55,087,066 |
