# Supplementary material for: DNA Methylation Profiling of Breast Cancer Cell Lines along the Epithelial Mesenchymal Spectrum—Implications for the Choice of Circulating Tumour DNA Methylation Markers
Source: Int J Mol Sci. 2018 Aug 28;19(9):2553. doi: 10.3390/ijms19092553 (PMC6164039; doi:10.3390/ijms19092553)
Supplement: Supplementary file 1 [file ijms-19-02553-s001.zip › Additional file 3_Table S2.docx]

**Table S2: Summary of PCR conditions for sixteen methylation-sensitive high-resolution melting (MS-HRM) assays**

|  | **REACTION MIXTURE** | | | **ACTIVATION** | **AMPLIFICATION** | | | | | **INACTIVATION** | **MELT** |
| --- | --- | --- | --- | --- | --- | --- | --- | --- | --- | --- | --- |
| **Genes** | **MgCl2  concentration  (mmol/L)** | **Forward primer  concentration  (nmol/L)** | **Reverse primer  concentration  (mmol/L)** | **95˚C  hold time  (min)** | **Number  of cycles** | **95˚C  cycling time  (sec)** | **Annealing  temperature  (celsius)** | **Annealing  cycling time  (sec)** | **72˚C  cycling time  (sec)** | **97˚C  hold time  (min)** | **Temperature  range (celsius),  0.2˚C/step** |
| ***AKR1B1*** | 2.5 | 200 | 200 | 15 | 55 | 10 | 61 | 20 | 30 | 1 | 70-95 |
| ***APC*** | 2.5 | 200 | 300 | 15 | 55 | 10 | 55 | 20 | 20 | 1 | 70-90 |
| ***BRCA1*** | 3 | 200 | 200 | 15 | 50 | 10 | 60 | 10 | 20 | 1 | 70-90 |
| ***CDH1*** | 2.5 | 200 | 200 | 15 | 55 | 10 | 63 | 10 | 20 | 1 | 70-95 |
| ***CDKN2A*** | 2.5 | 200 | 200 | 15 | 55 | 10 | 60 | 20 | 20 | 1 | 72-90 |
| ***CRABP1*** | 2.5 | 200 | 200 | 15 | 50 | 10 | 57 | 20 | 20 | 1 | 70-95 |
| ***DKK3*** | 1.5 | 400 | 400 | 15 | 55 | 10 | 66 | 20 | 20 | 1 | 72-90 |
| ***EGFR*** | 2.5 | 300 | 300 | 15 | 55 | 10 | 56 | 20 | 30 | 1 | 70-95 |
| ***GRHL2*** | 3 | 300 | 300 | 15 | 55 | 10 | 63 | 20 | 20 | 1 | 70-90 |
| ***MIR200C*** | 3 | 200 | 200 | 15 | 50 | 10 | 57 | 20 | 20 | 1 | 70-95 |
| ***RARß*** | 3 | 200 | 300 | 15 | 55 | 10 | 69 | 20 | 20 | 1 | 70-90 |
| ***RASSF1A*** | 2.5 | 400 | 400 | 15 | 55 | 10 | 58 | 20 | 20 | 1 | 68-90 |
| ***SFRP2*** | 2.5 | 200 | 200 | 15 | 55 | 10 | 59 | 20 | 30 | 1 | 65-95 |
| ***GFRA1*** | 2.5 | 200 | 200 | 15 | 55 | 10 | 65 | 20 | 30 | 1 | 65-95 |
| ***VIM*** | 2.5 | 200 | 300 | 15 | 55 | 10 | 56 | 20 | 20 | 1 | 68-90 |
| ***TWIST1*** | 2.5 | 200 | 300 | 15 | 55 | 10 | 55 | 20 | 20 | 1 | 72-90 |
