# Supplementary material for: DNA Methylation Profiling of Breast Cancer Cell Lines along the Epithelial Mesenchymal Spectrum—Implications for the Choice of Circulating Tumour DNA Methylation Markers
Source: Int J Mol Sci. 2018 Aug 28;19(9):2553. doi: 10.3390/ijms19092553 (PMC6164039; doi:10.3390/ijms19092553)
Supplement: Supplementary file 1 [file ijms-19-02553-s001.zip › Additional file 4_Table S3.docx]

**Table S3**: **Sequences before bisulfite treatment, sequences to analyse and dispensation order for tested bisulfite pyrosequencing assays.**

| Genes | Types of sequences | Sequences |
| --- | --- | --- |
| *AKR1B1* | Sequence before bisulfite treatment | CCTTCGCGCAGCGGCGCGCCAACCGCAGGCGCCCTTTCTGCCGACCTCACGGGCTATTTAAAGGTACGCGCCG |
|  | Sequence to analyse | TTTTYGYGTAGYGGYGYGTTAATYGTAGGYGTTTTTTTTGTYGATTTTAYGGGTTATTTAAAGGTAYGYGTYG |
|  | Dispensation order | **G**T**T**C**TA**GTCG**C**TA**T**GTC**A**GTC**A**GTCGT**G**ATC**T**GTA**T**GTCGT**TA**GTC**T**GAT**TG**ATCGTATAGT**G**ATC**A**GTC**A**GTC**T** |
| *CRABP1* | Sequence before bisulfite treatment | CGCCCCCAACCCCGCCGGGCTTCTGGCCCCGCCCCGCCGGCCCTGCCCCCGCTCCCCCGCAGCCCCTACTCGGTATGCGCTC |
|  | Sequence to analyse | YGTTTTTAATTTYGTYGGGTTTTTGGTTTYGTTTYGTYGGTTTTGTTTTYGTTTTTTYGTAGTTTTTATTYGGTATGYGTTT |
|  | Dispensation order | **A**TCG**C**T**TG**AT**T**C**TA**GTC**T**GT**TA**GT**T**C**TA**GT**T**C**TA**GTC**T**GT**TA**GT**T**C**TA**GT**T**C**T**GTAGT**TG**ATC**T**GTAT**A**GTCG |
| *GFRA1* | Sequence before bisulfite treatment | TCGGGGAGACCGAGTTTGAATCAAATCTGCGTGCGCCCAGCTGTCAAATCTGCAAACCTATCACGCCAGACAATGGGCCGCCGCGGAGGA |
|  | Sequence to analyse | TYGGGGAGATYGAGTTTGAATTAAATTTGYGTGYGTTTAGTTGTTAAATTTGTAAATTTATTAYGTTAGATAATGGGTYGTYGYGGAGGA |
|  | Dispensation order | GTCGGAGTATCTGAGTGAT**C**ATAGTCGTAGTCGTAGTGTATGTATATGATCGTAGATATAGTCTAGTCTAGTCGAG |
| *GRHL2* | Sequence before bisulfite treatment | CGATCCAGGAGGACTCCGCGCCGCCCGGCCGCCTCCGAGCTCGGG |
|  | Sequence to analyse | YGATTTAGGAGGATTTYGYGTYGTTYGGTYGTTTTYGAGTTYGGG |
|  | Dispensation order | **G**TCGATAGAG**T**AT**T**C**TA**GTC**A**GTC**TA**GTC**TA**GTC**TA**GT**T**C**T**GA**T**GTC**T** |
| *RARB* | Sequence before bisulfite treatment | CGAGAACGCGAGCGATCC |
|  | Sequence to analyse | YGAGAAYGYGAGYGATTT |
|  | Dispensation order | **G**TCGAG**T**ATC**A**GTCGA**T**GTCGATTC |
| *RASSF1A* | Sequence before bisulfite treatment | CATTGCGCGGCTCTCCTCAGCTCCTTCCCGCCGC |
|  | Sequence to analyse | TATTGYGYGGTTTTTTTTAGTTTTTTTTYGTYGT |
|  | Dispensation order | **GC**TAT**A**GTC**A**GTCGT**T**A**T**GT**T**C**TA**GTC**T**G |
| *SFRP2* | Sequence before bisulfite treatment | CGGCTCATTCTGCTCCCCCGGGTCGGAGCCCCCCGGAGCTGCGCGCGGGCTTGCAGCGCCTCGCCCGCGCTGTCCTCCCGGTGTCCCGCTTCTCCGCGCCCCAGCCGCCGGCTGC |
|  | Sequence to analyse | YGGTTTATTTTGTTTTTTYGGGTYGGAGTTTTTYGGAGTTGYGYGYGGGTTTGTAGYGTTTYGTTYGYGTTGTTTTTTYGGTGTTTYGTTTTTTYGYGTTTTAGTYGTYGGTTGTT |
|  | Dispensation order | GTCGTATTAGTTCTAGTCGATGTTCTGAG**C**TAGTCAGTCAGTCGTGTATGTCAGTTCTAGTCTAGTCGTAGTTCTGTAGTTCTAGTTCTAGTCGTTATGTCTAGTCTGTG |

*bold nucleotides in each dispensation order are control nucleotides added by the advanced software (Bio Molecular Systems, v2.0.11), which allow the detection of incomplete bisulfite conversion or non-specific products.
